# Lip-Lick Cheilitis and Its Connection to the Brain

**DOI:** 10.7759/cureus.64312

**Published:** 2024-07-11

**Authors:** Riley J Stone, Gianna M Labert, Robert A Norman

**Affiliations:** 1 Anthropology, University of South Florida, Tampa, USA; 2 Allied Health, University of Tampa, Tampa, USA; 3 Dermatology, Nova Southeastern University Dr. Kiran C. Patel College of Osteopathic Medicine, Fort Lauderdale, USA

**Keywords:** prevention, stress, dermatitis, lip licking, psychocutaneous

## Abstract

An eight-year-old male who habitually licked his lips and presented with dry, flaky, and red skin bordering the outside of his lips was diagnosed with lip-lick cheilitis. This condition is exacerbated or, at times, caused by chronic lip-licking, leading to irritation and discomfort. Treatment included tacrolimus 0.03% topical ointment, which he was instructed to apply twice daily. He was also advised to stop licking his lips, maintain proper hydration, and use a stress ball when tempted to lick the irritated region. Following these interventions, there was a clear reduction in both irritation and redness. This condition frequently reflects psychocutaneous interactions, commonly observed in patients with underlying psychological stress, displaying the connection between the brain and skin.

## Introduction

Lip-lick cheilitis, also known as lip-licker’s dermatitis, is a condition caused by the patient’s chronic habit of licking their lips, allowing for saliva to damage the skin. The clinical features include scaliness, dryness, persistent redness, and ongoing inflammation on the vermilion border [1]. The condition commonly occurs under poor climate conditions, such as cold or dry weather. In an attempt to relieve the dryness and irritation, the patient wets their lip region with saliva, resulting in a breakdown of the protective barrier caused by the digestive enzymes in saliva [1,2]. Furthermore, with the steady continuation of the chronic habit, more severe conditions can develop, including cheilitis simplex, angular cheilitis, factitial cheilitis, or secondary infections [2]. 

Cheilitis simplex, also known as chapped lips, is a condition that involves scaly, fissured, and flaky lips. Cheilitis simplex is commonly a result of harsh weather conditions or habitual lip-licking. Typically, cheilitis simplex appears on the lower lip, presenting as irritated yellow, red, crusted lesions. Contact dermatitis relays how external exposures and products can amplify the irritation of the site [3]. For example, symptoms can further be exacerbated by acne products [2]. In fact, recreational and occupational exposures, such as musicians with wind instruments or snorkelers, can intensify symptoms by irritating the affected site, leading to contact dermatitis [2].

Angular cheilitis is a condition resulting in cracked, irritated sores on the lips. This condition involves the oral commissures of the lips, also known as the corners of the mouth where the upper and lower lips meet. Excessive salivation of the lips, medications, other medical conditions, and nutritional deficiencies can be linked to macerations, pruney-textured skin over-exposed to water, of the corners of the lips [2]. Patients with medical conditions, including Irritable Bowel Disease (IBD) or diabetes, are more susceptible to developing secondary infections from angular cheilitis [2]. Additionally, angular cheilitis is connected to bulimia, as purging can reduce saliva production, causing flaky, dry lips, which are susceptible to developing angular cheilitis and secondary infections [4].

Factitial cheilitis is caused by constant lip-licking behaviors, lip biting, and picking of the lips [5]. When left untreated, a crusty, yellow keratinaceous build-up is formed, and ulcerations around the affected site may occur [5]. In pediatric patients and young women with a psychological history of anxiety, factitious cheilitis is common [2]. Because factitious cheilitis often involves an underlying psychological disorder, treatment must involve a mixture of behavioral intervention and any necessary topical treatments [2]. It is vital to design an effective treatment regimen for patients presenting with lip-licking dermatitis. Without proper treatment, the irritation and flakiness of the skin will worsen, allowing bacteria such as *Staphylococcus aureus* or *Candida albicans* to infect the site [5]. These types of microorganisms thrive in compromised skin, which allows for colonization. When this occurs, the symptoms will worsen with the potential for systemic spread, infecting multiple systems throughout the body. 

There are several reasons a patient might be inclined to lick their lips, including abnormal weather conditions, sunburn, anxiety, atopic dermatitis, and severe nasal congestion [2]. Patients with systemic diseases, such as lupus erythematosus, Crohn’s disease, and sarcoidosis, are also more susceptible to developing lip-licking behaviors, as dry lips are a common symptom of these conditions [6].

## Case presentation

**Figure 1:**
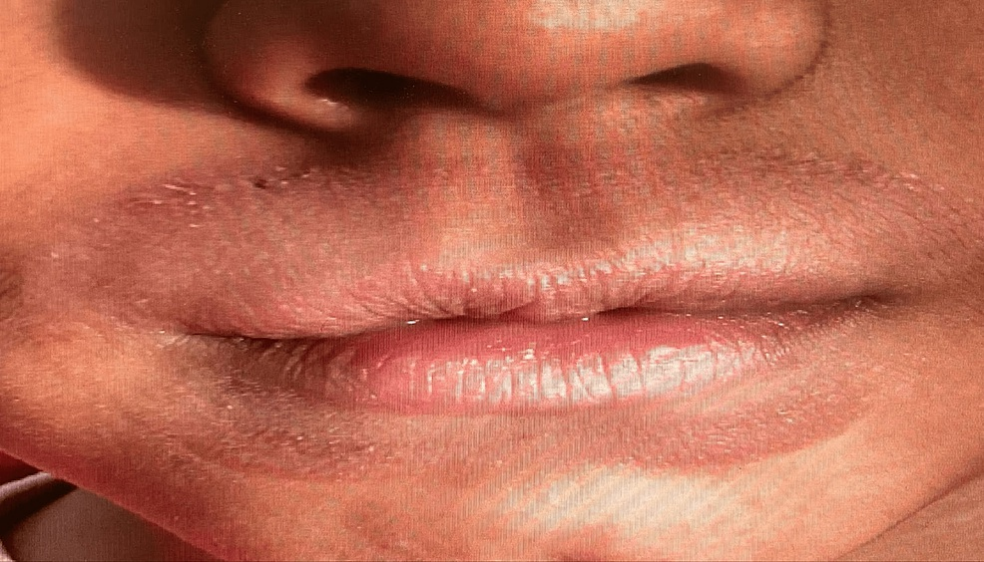
Erythema around the lips

**Figure 2:**
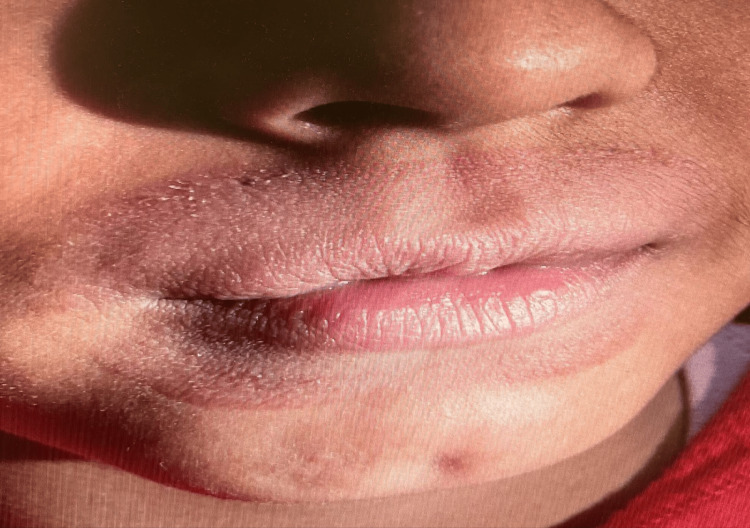
Close-up of irritation and dryness around the lips

An eight-year-old male presented to the dermatologist with a halo-like red, itchy rash around the perimeter of his lips, expanding toward his nostrils and down to the middle of his chin (Figures 1-2). The symptoms included erythema around his lip region, the urge to lick his lips to temporarily “alleviate” dryness, and flaking skin, especially atop his upper lip. The patient had been previously prescribed hydrocortisone, which was ineffective in alleviating his symptoms.

It was evident the patient’s lip-licking was constant, especially during the driest period of the year. The patient also declared they were stressed due to school responsibilities, indicating that anxiety is most likely playing a role in the condition. For effective treatment, the patient was advised to stop licking his lips, maintain proper hydration, and utilize a stress ball when tempted to lick his lip region. The patient was prescribed tacrolimus 0.03% topical ointment with instructions to apply twice daily to irritated areas around the lips. 

**Figure 3:**
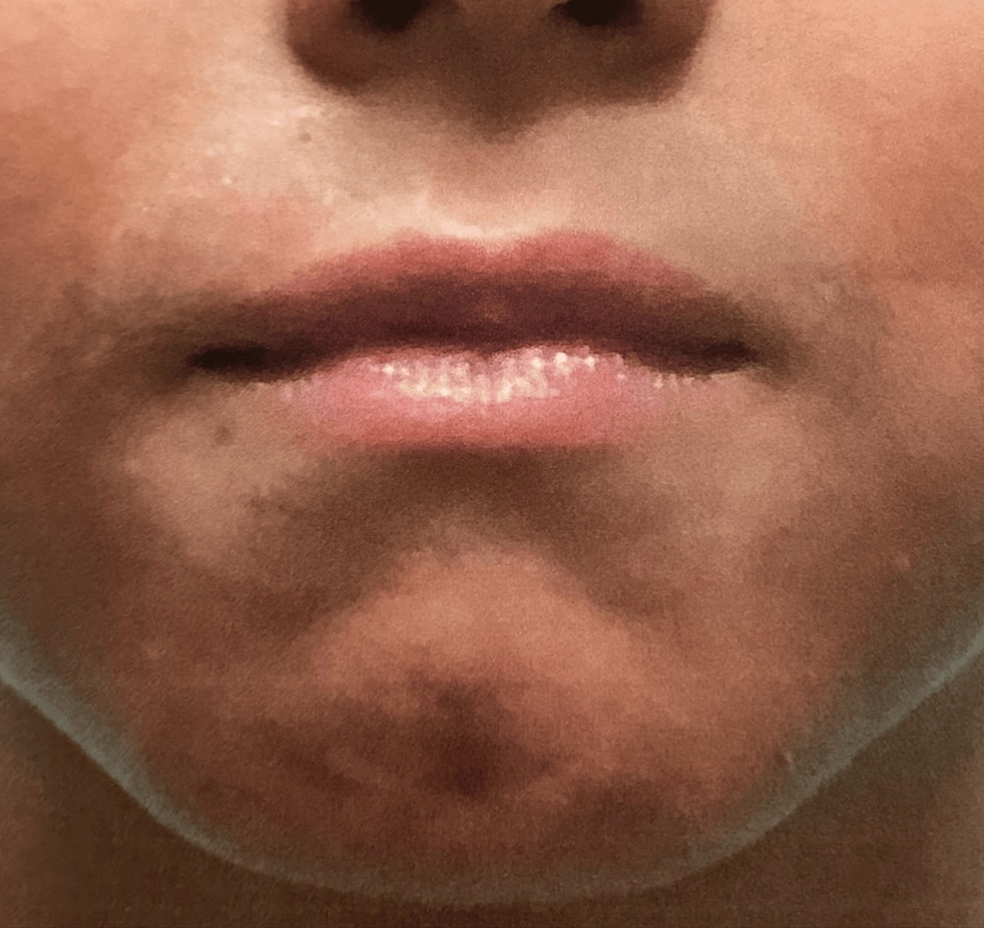
Lips after treatment

This treatment plan remained consistent for two additional follow-up appointments, each four weeks apart, making the duration of the treatment eight weeks. Throughout the span of these appointments, there was consistent relief in his symptoms with occasional flare-ups. Within two months of his initial diagnosis, the patient had significantly reduced his lip-licking behavior. Although his lips remained dry, the irritation had dissipated, and the erythema decreased significantly (Figure 3). The patient was advised to discontinue the use of tacrolimus 0.03% topical ointment and to apply Aquaphor as needed.

## Discussion

We present an eight-year-old male with a ring of red, flaky, and irritated skin encompassing his lip region, expanding toward his nose and down to the middle of his chin. Upon examination, he was diagnosed with lip-lick cheilitis. This was due to the patient exhibiting chronic irritation in the areas around the lips from the licking behavior. Therefore, the diagnosis was apparent because the location is exclusively around the lips where saliva frequently contacts the skin. The patient was prescribed 0.03% tacrolimus topical ointment and instructed to apply it twice daily for a total of eight weeks. Lip-lick cheilitis is the center of this study as it can often arise in patients with underlying conditions or psychological stress. 

Although the tongue is the culprit of lip-lick cheilitis, it is important to consider the potential causes of this chronic habit. Dermatologists must determine whether any other coexisting health problems can possibly be contributing to the affected skin site. Therefore, it is imperative to address both behavioral concerns and physical concerns.

Patients with anxiety may unconsciously lick their lips as a stress response, often without realizing they are engaging in such behavior during moments of heightened anxiety [6]. In such cases, behavioral interventions are often necessary to break the habit. Moreover, among pediatric patients and young women with a psychological history, many of the differential diagnoses previously discussed - such as factitious cheilitis, angular cheilitis, and cheilitis simplex - are frequently observed [7]. These conditions often carry a psychosomatic component, where emotion or psychological stress can trigger the physical symptoms. This further highlights the importance of treating the patient as a whole, instead of simply the condition, to effectively address all underlying causes.

To effectively treat lip-lick cheilitis with a psychosomatic component, patients must receive a necessary combination of both dermatological treatment and psychological intervention. Psychological intervention would involve cognitive behavioral therapy (CBT), a form of psychotherapy targeting the triangle of behavior, feelings, and thoughts [8]. The patient must learn to identify the interrelationship between their thoughts and behaviors, which can be a key factor in managing symptoms [8].

At the beginning of CBT, the patient will relay their struggles and discuss their therapy goals [8]. In the case of a patient with lip-lick cheilitis, the patient would explain how their lip-licking behavior is attributed to their thoughts [8]. From there, the therapist or psychologist will work to help the patient neutralize the triangle of behavior, feelings, and thoughts with the accompaniment of relaxation techniques. For example, an individual's anxiety in a public setting may make them think, “Why is everyone looking at me? Do I look silly?” As a conditioned response, the individual may resort to licking their lips as a coping mechanism. However, with CBT, the individual would learn to neutralize this thought, substituting it with a more neutral reflection, thus reducing the conditioned response of lip-licking. Through practice, patients can experience significant improvements in both skin conditions and mental health outcomes [9].

Because the patient of this study had no reports of psychological history, it was evident lip-lick cheilitis was a direct result of consistent lip-licking, with a possible linkage to anxiety from school. Due to the digestive enzymes in saliva, the delicate skin of the lips became vulnerable. As the presented patient continued this habit while enduring environmental factors such as a dry climate and ultraviolet exposure, this type of contact dermatitis was given the necessary conditions to develop [7].

If lip-licking is found to be secondary to another medical condition, such as IBD or diabetes, the physician prescribing the medication to treat those conditions must be notified. In addition, it is good practice for patients experiencing cracked, red lips to visit a dermatologist to ensure there is no lip dermatitis or secondary infections of the affected site.

Hydrocortisone was likely ineffective for the patient due to the damage and sensitivity of the skin barrier, a consequence often worsened by this medication. This corticosteroid is commonly known to decrease thickness by reducing collagen synthesis, which can worsen the already damaged skin barrier [10]. In cases like this one, where the patient is presenting not only irritation but also lacking a significant amount of moisture, the choice treatment must have a dual effect of preventing inflammation and healing the dry skin [11]. Therefore, a medication such as tacrolimus possesses both those qualities and does not have as many side effects as hydrocortisone [12]. The transition from hydrocortisone to tacrolimus underscores the importance of selecting a treatment specific to the pathophysiologic aspects of the condition being addressed.

However, it is important to keep the patient on the necessary prescriptions only as needed. Prolonged use of tacrolimus or inadequate dosing is correlated with an increased risk of lymphoma and cancer [13]. This is because topical ointment diminishes the immune system, thereby increasing the vulnerability of developing cancers [13].

## Conclusions

We present a case that involves a young male who developed lip-lick cheilitis, influenced by both environmental factors and a chronic habit with an unknown etiology. This case examines a situation where tacrolimus proved effective and topical hydrocortisone proved ineffective. The successful treatment shows the importance of understanding diverse immunomodulatory therapies for dermatitis. Furthermore, this case emphasizes the need for exploring psychodermatological interactions, as insights into preventive measures and a more interdisciplinary approach can be utilized to treat brain-skin interactions effectively.
